# An unusual Wolf’s isotopic response: myelodysplastic syndrome with acute myeloid leukemia developed on herpes zoster lesions^⋆^

**DOI:** 10.1016/j.abd.2023.02.011

**Published:** 2024-02-22

**Authors:** Yu Zhu, Wei Wu

**Affiliations:** Department of Dermatovenereology, Affiliated Hospital of Guangdong Medical University, Zhanjiang, Guang Dong, China

Dear Editor,

A 67-year-old male visited our clinic, complaining of severely itchy and tufted papules on his right waist and abdomen for more than 6 months. More than 6 months ago, cluster erythema blisters appeared on the right waist and abdomen which were diagnosed as “herpes zoster” clinically. Treated with traditional Chinese medicine for 10 days, erythema and blisters disappeared. Later on metameric papules, and plaques appeared on the healing herpes zoster with intense pruritus. His medical history was otherwise unremarkable. Physical examination: vital signs are stable; cardiopulmonary and abdominal examination was normal. Enlarged lymph nodes were palpable in the cervical and axillary regions, bilaterally. Metameric violaceous papules and plaques with infiltration were seen on the right waist and abdomen, corresponding to the right dermatomes of T8‒T10 (Fig. 1 A). Histopathology (Fig. 2 A‒B): lymphoid cells infiltrate in the epidermis and hair follicles, diffuse mild atypia lymphocytes, and epithelioid cells infiltrate in the whole dermis and subcutaneous fat layer. Immunohistochemistry: CD20, Myeloperoxidase (MPO), and CD117 were negative (Fig. 3 A‒C). CD3, CD4, and CD8 were positive (CD4+ are more than twice CD8+ cells), and above 30% cells showed a positive staining with Ki67 (Fig. 4 A‒D). Axillary lymph node puncture : lymph node inflammation. The patient refused lymph node biopsy. Cutaneous T-cell lymphoma was considered initially. However, he also had a persistent decrease in leukocyte count and moderate anemia. Further tests were performed to clear if hematological disorders could be associated. Electrophoresis of serum proteins revealed a rise in gamma globulin and no abnormal globulin M band . Bence Jones protein in urine was negative. Eventually, myelodysplastic syndrome with Acute Myeloid Leukemia (AML) was diagnosed by the results of bone marrow and gene tests (mutation of DNMT3A, IDH2, STAG2, and ASXL1). He was then transferred to the Department of Hematology to receive chemotherapy with azacitidine, daunorubicin and cytarabine. After one week of chemotherapy, the patient's pruritus was significantly relieved and the lesions were significantly improved. After four months of chemotherapy, his rash had improved. (Fig. 1 B). In addition, his blood changes and bone marrow images returned to normal, and the enlarged lymph nodes disappeared. The infiltration of tumor cells in the epidermis and the positive for markers of T-lymphocyte, especially the TH4 markers are dominant, with the absence of myeloid antigens observed in skin biopsies and immunohistochemistry respectively, led the misdiagnose of Mycosis Fungoides (MF) initially. Paradoxically, his rash is in accord with the plaque stage of MF, but the atypia lymphocytes infiltrate in the whole dermis and subcutaneous fat layer observed in the histopathology could be a tumor stage of MF. Moreover, the disease course is only 6 months, so the diagnosis of MF was ruled out. Given the positive effects of chemotherapy directed at AML, we consider that the metameric skin manifestation is cutaneous metastasis of myelodysplastic syndrome with acute myeloid leukemia in healed herpes zoster, corresponding Wolf's Isotopic Response (WIR).

**Figure 1:**
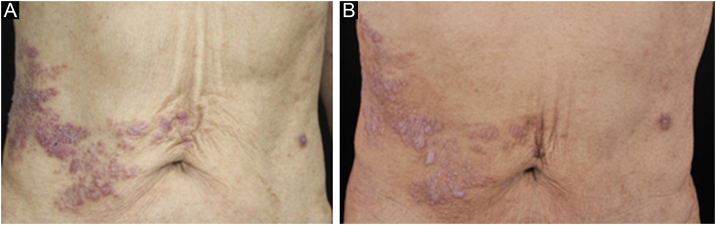
(A) Metameric dark red papules, plaques with infiltration on the right waist and abdomen, corresponding to the right dermatomes of T8‒T10. (B) After 4-months of chemotherapy: infiltration becomes flatter and lighter than before.

**Figure 2:**
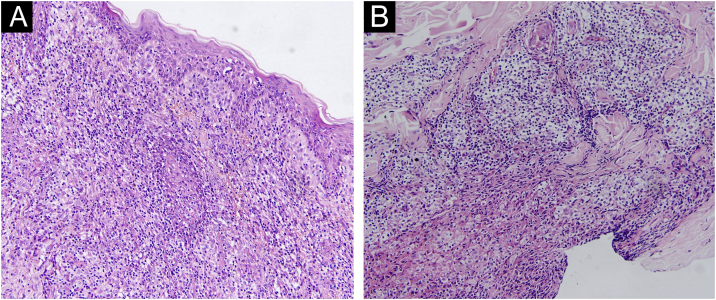
(A) Lymphoid cell infiltration in the epidermis and hair follicles, (B) Diffuse lymphocytes and epithelioid cell infiltration in the whole dermis. (Hematoxylin & eosin, ×200).

**Figure 3:**
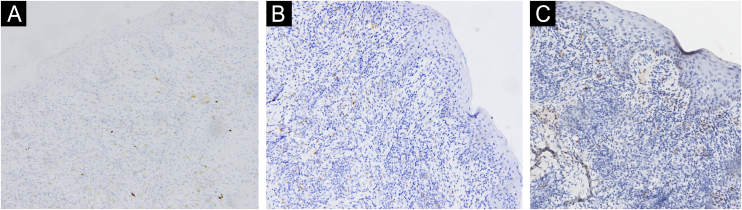
Negative immunohistochemical staining for CD20 (A), Myeloperoxidase (MPO) (B) and CD117 (C) (×200).

**Figure 4:**
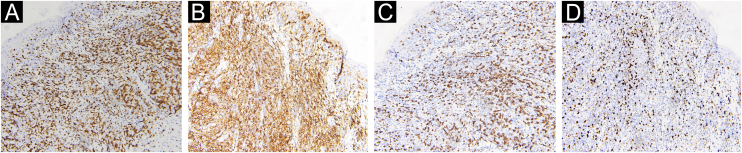
Positive immunohistochemical staining for CD3 (A), CD4 (B), and CD8 (C), Ki 67 was positive in more than 30% of the cells (D) (×200).

WIR describes the occurrence of a new skin disorder at the site of another unrelated and already healed skin disease. Herpes infection is the most common first disease, especially herpes zoster. The second diseases at the same site include granulomatous and lichenoid reactions, leukemia cutis, skin tumors, and infections.^1^ Cutaneous metastasis of myeloid leukemia following herpes infection is pretty infrequent, compared with lymphoid leukemia, only one case has been reported until now.^2^ The previous report is leukemia cutis developing as a WIR in a case of treatment‑refractory AML, but the case we reported is WIR preceding the discovery of myelodysplastic syndrome with acute myeloid leukemia. Moreover, myeloid leukemia cutis at the site of striae distensae^3^ and sarcoid tissue reaction on herpes zoster scars in myelodysplastic syndrome patients^4^ have also been reported. When new skin lesions appear at the site of healed herpes zoster accompanied by peripheral blood abnormality, a biopsy of the skin and bone marrow is required to rule out potential hematological tumors. The case we reported is an unusual WIR, with cutaneous metastasis of myelodysplastic syndrome with acute myeloid leukemia s. We are unable to find any similar previous report.

## Financial support

None declared.

## Authors' contributions

Yu Zhu: Contribution with the preparation and writing of the manuscript.

Wei Wu: Contribution to manuscript critical review, and approval of the final version of the manuscript.

## Conflicts of interest

None declared.
